# Association of Child and Adolescent Mental Health With Adolescent Health Behaviors in the UK Millennium Cohort

**DOI:** 10.1001/jamanetworkopen.2020.11381

**Published:** 2020-08-10

**Authors:** Erin Hoare, Andre O. Werneck, Brendon Stubbs, Joseph Firth, Sam Collins, Kirsten Corder, Esther M. F. van Sluijs

**Affiliations:** 1 Medical Research Council, Epidemiology Unit, Centre for Diet and Activity Research, University of Cambridge, Cambridge, United Kingdom; 2 The Institute for Mental and Physical Health and Clinical Translation, Food and Mood Centre, Barwon Health, Deakin University, School of Medicine, Geelong, Australia; 3 Department of Physical Education, Universidade Estadual Paulista “Júlio de Mesquita Filho,” Presidente Prudente, Brazil; 4 Institute of Psychiatry, Psychology and Neuroscience, Department of Psychological Medicine, King's College London, London, United Kingdom; 5 Physiotherapy Department, South London and Maudsley NHS Foundation Trust, Denmark Hill, London, United Kingdom; 6 Division of Psychology and Mental Health, University of Manchester, Manchester, United Kingdom; 7 NICM Health Research Institute, Western Sydney University, Westmead, Australia; 8 Centre for Youth Mental Health, University of Melbourne, Melbourne, Australia

## Abstract

**Importance:**

There is potential for mental health status to act as a determinant of an individual’s ability to engage in healthful lifestyle behaviors.

**Objective:**

To investigate the associations of parent-reported mental health problems during childhood and self-reported mental health problems in adolescence with health behaviors in adolescence.

**Design, Setting, and Participants:**

This cohort study used data from wave 4 (collected in 2008) and wave 6 (collected in 2015) of the Millennium Cohort Study, a UK population-representative longitudinal study of young people born during 2000 to 2001. Wave 4 included data on parent-reported mental health issues for children at age 7 years. Wave 6 included data on self-reported mental health problems as well as health behaviors for the same children at age 14 years. Data were analyzed July 5, 2020.

**Exposures:**

Mental health problems at age 7 years were parent-reported using Strengths and Difficulties Questionnaire. Mental health problems at age 14 years were self-reported using the Short Mood and Feelings Questionnaire.

**Main Outcomes and Measures:**

Health behaviors at age 14 years were the main outcome of interest. Sleep duration; fruit, vegetable, and soft drink consumption; and social media use were self-reported using recall on a typical day. Regression models were calculated for each lifestyle variable, with mental health change from ages 7 to 14 years as the exposure variable. Data were weighted to account for the potential clustering of region of sampling and adjusted for nonresponse.

**Results:**

A total of 9369 participants were included in waves 4 and 6 of the Millennium Cohort Study, including 4665 (48.1%) girls and 6014 participants (81.9%) who were born in England. Adolescents who self-reported mental health problems at age 14 years only were less likely to have at least 9 hours of sleep (odds ratio [OR], 0.39; 95% CI, 0.34-0.45) and to consume fruit (OR, 0.55; 95% CI, 0.46-0.65) and vegetables (OR, 0.66; 95% CI, 0.52-0.83) reported greater use of social media (*b* = 0.62; 95% CI, 0.49-0.75) compared with individuals who did not have mental health problems at both time points. Similarly, those with mental health problems at both time points were less likely to achieve 9 hours sleep (OR, 0.68; 95% CI, 0.51-0.90), consume fruit (OR, 0.39; 95% CI, 0.26-0.58) and vegetables (OR, 0.57; 95% CI, 0.35-0.91), and reported greater social media use (*b* = 0.63; 95% CI, 0.34-0.91).

**Conclusions and Relevance:**

These findings suggest that the presence of depressive symptoms at ages 7 and 14 years and at age 14 years only were associated with some health behaviors in adolescence. These findings are particularly important given that independent health behaviors can deteriorate and become habitual during adolescence, and adolescence is a known time for the first emergence of mental health problems that continue into adulthood.

## Key Points

**Question:**

Are mental health problems during childhood and adolescence associated with health behaviors during adolescence?

**Findings:**

In this cohort study including 9369 participants, adolescents with self-reported mental health problems at both ages 7 years and 14 years and at age 14 years only were less likely to have at least 9 hours of sleep and to consume fruit and vegetables and reported greater use of social media compared with individuals who had no parent-reported mental health problems at age 7 years or self-reported problems at age 14 years.

**Meaning:**

These findings suggest that mental health during childhood and adolescence was associated with health behavioral outcomes, and this should be considered in future efforts to improve health behaviors and in noncommunicable disease prevention efforts.

## Teaser

This cohort study examines associations of reported mental health issues at ages 7 and 14 years with health behaviors in adolescence.

## Introduction

Adolescence and young adulthood, the age periods from 10 to 24 years, are significant transitional life periods with known unique risks for the development of mental health problems.^1^ A 2019 study^2^ estimated that 9% of British adolescent boys experienced depressive symptoms. This proportion was 24% among British adolescent girls. The factors associated with risk of or protection from mental health disorders are understood to be influenced by individual, social, and environmental factors.^3^

Although lifestyle behaviors have been recognized as novel targets for prevention of mental health conditions, there is also potential for mental health status to act as a determinant of an individual’s ability to engage in healthful lifestyle behaviors. As an example, depression is characterized by low motivation, low energy, and loss of pleasure in daily activities, and this has been found to be associated adverse outcomes in daily behaviors, such as dietary choices, physical activity, and sleep.^4,5^ This potentially reciprocal association holds important public health implications, particularly regarding the highly elevated rates of cardiometabolic comorbidities associated with poor mental health.^6^

Importantly, evidence also points to lifestyle behaviors, such as sleep and diet, for the promotion of positive mental health.^5^ Lifestyle behaviors are appealing targets, given that they are largely modifiable, unlike many other risk factors for mental disorders. There is also great potential for population reach and accessibility, and these factors may carry additional physical health benefits, such as noncommunicable disease prevention.^7^ Social media use has emerged as a highly prevalent behavior among young people and has been associated with internalizing and externalizing problems,^8^ although its association with mental health has been debated.^9^ Furthermore, there is an increasing interest in sleep as a health behavior, which is crucial for healthy development in adolescents.^5,10^

While lifestyle behaviors have been assessed as factors associated with mental health, there is an imperative to understand the association of mental health problems in childhood and adolescence with engagement in health behaviors during adolescence. Therefore, the aim of this study was to investigate the associations of parent-reported mental health during childhood and self-reported mental health problems during adolescence with subsequent diet, sleep, and social media use behaviors at age 14 years among a large, nationally representative cohort of young people in the UK. We hypothesized that owing to the symptoms and experiences that characterize depression and mental health problems generally, worse mental health problems at ages 7 and 14 years would be associated with adverse health behaviors at age 14 years.

## Method

All procedures involving human participants were approved by Northern and Yorkshire Multi-Centre Research Ethics Committee of the National Health Service. Written informed consent was provided from all participating families. All procedures contributing to this work comply with the Declaration of Helsinki of 1975, as revised in 2008.^11^ This study is reported following the Strengthening the Reporting of Observational Studies in Epidemiology (STROBE) reporting guideline for cohort studies.

### Sample

The Millennium Cohort Study (MCS) is a national longitudinal birth cohort study conducted among young people born in the UK between September 2000 and January 2002. The first wave of data collection occurred during 2001 to 2002 (ie, wave 1) with 5 follow-ups released to date, occurring in 2003 to 2004 (ie, wave 2), 2006 (ie, wave 3), 2008 (ie, wave 4), 2012 to 2013 (ie, wave 5), and 2015 to 2016 (ie, wave 6). This study used data from wave 4, when participants were aged approximately 7 years, and wave 6, when participants were aged approximately 14 years. Waves 4 and 6 were selected owing to the availability of comprehensive health behavioral and mental health measures across 2 time points. Further details on the MCS have been reported elsewhere.^12^

### Measures

#### Outcome

Health behaviors at age 14 years, including sleep; fruit, vegetable, and soft drink consumption; and social media use, were the outcome of interest. Sleep was estimated in hours based on responses to 2 questions: “What time do you typically go to bed on a school night?” and “What time do you typically wake on a school morning?” Responses were converted into hours and categorized into whether participants met or did not meet age-specific sleep recommendations of 9 hours of sleep per night.^13^ Fruit, vegetable, and soft drink consumption and social media use were self-reported. Fruit and vegetable consumption were measured using the question “How often do you eat at least 2 portions of [fruit/vegetables]?” with responses never, some days but not all days, and every day. Soft drink consumption was measured by the question “How often do you drink sweetened drinks?” with responses classified into less than once per week, 1 to 2 days per week, 3 to 6 days per week, and every day. The 3 diet variables (ie, fruit, vegetable, and soft drink consumption) were dichotomized into consumers and nonconsumers. Social media use was measured by asking participants “How many hours per week do you spend on social networking sites?” with responses less than half an hour, half hour to less than 1 hour, 1 hour to less than 2 hours, 2 hours to less than 3 hours, 3 hours to less than 5 hours, 5 hours to less than 7 hours, and 7 hours or more, and the variable was treated as continuous.

#### Exposure

Parent-reported mental health problems at age 7 years and self-reported mental health problems at age 14 years were the exposure variables of interest. At age 7 years (ie, wave 4), parents completed the 25-item Strengths and Difficulties Questionnaire (SDQ) to assess child mental health problems.^14^ The SDQ comprises 5 domains measured with 5 items each: emotional symptoms (eg, many fears, easily scared), peer problems (eg, gets on better with adults than with other children), conduct problems (eg, often lies or cheats), hyperactivity (eg, restless, overactive, cannot stay still for long) and prosocial behavior (eg, shares readily with other children [reverse scored]). For each item, 0 is given if the response is not true; 1, somewhat true; and 2, certainly true. Of the difficulties items (excluding prosocial behavior) a total score of 14 or greater was indicative of mental health problems, per author recommendations.^15^ The SDQ has demonstrated good reliability and validity in population-based health surveys.^16^

Depressive symptoms at age 14 years (ie, wave 6) were self-reported using the Short Mood and Feelings Questionnaire (SMFQ),^17^ a 13-item self-report questionnaire designed to assess depressive symptoms. The measure asks “For each question, please check how much you have felt or acted in this way [during] the past two weeks,” with available responses true, scored as 2; sometimes, 1; or not true, 0. Example items include “I felt miserable or unhappy” and “I found it hard to think properly or concentrate.” Items are scored and summed to give a final overall score ranging from 0 to 26, with higher scores indicating greater presence of depressive symptoms. The SMFQ has been validated in clinical and nonclinical populations.^18,19^ We adopted a clinical cutoff score of 8 or greater to indicate presence of depressive symptoms, which corresponds to previous research that demonstrated content validity of the SMFQ in relation to the *International Statistical Classification of Diseases and Related Health Problems, Tenth Revision* for a diagnosis of depressive episode.^19,20^ To assess change in mental health problems over time, we used the dichotomized data to create a categorical variable defined as (1) no mental health problems at either time point (reference category), (2) parent-reported mental health problems at age 7 years only, (3) self-reported mental health problems at age 14 years only, and (4) parent-reported mental health problems at age 7 years and self-reported mental health problems at age 14 years.

### Confounders

Confounders were selected based on their established evidence base for their association with the variables of interest in previous literature^21,22,23^ and were taken from wave 6 data, as they were assumed to be time invariant. Sex was self-reported. Household income was self-reported in the parent or guardian questionnaire at wave 6. Further details on data collection methods have been published elsewhere.^12^

### Statistical Analysis

All analyses were conducted in Stata statistical software version 15.1 (StataCorp). The data were weighted to account for the unequal selection probability and nonresponse at wave 6, per guidelines for analyzing the MCS. Weighting values from wave 6 were deemed most appropriate, given the sample was selected based on outcome variables collected at the age 14 years point; therefore participants who did not take part in this data collection wave were ineligible. The clustering of the MCS sample in terms of region of recruitment was taken into account by using the *svy* command. Specifically, the Stata command *svyset SPTN00 [pweight = FOVWT2], strata(PTTYPE2) fpc(NH2)* was used, whereby *SPTN00* is the fieldwork point number, *FOVWT2* is the overall weight for UK population accounting for unequal selection probabilities of wards and adjusted for nonresponse, *PTTYPE2* is the stratum within country, and *NH2* is the population correction factor. Participant characteristics were calculated in raw (ie, unweighted) values and weighted proportions for categorical variables or as weighted means and SDs for continuous variables, as appropriate. Regression models were calculated for each outcome lifestyle variable, with linear regression used for social media use and logistic regression for dichotomous variables (ie, sleep and fruit, vegetable, and soft drink consumption). Mental health status in childhood and adolescence, as the exposure variable, was a categorical variable (ie, no reported mental health problems at either time point [reference group] vs mental health problems at age 7 years only vs mental health problems at age 14 years only vs mental health problems reported at both time points). All models included the stratum design variable (ie, *PTTYPE2*) as a dummy variable per MCS guidelines to account for sampling stratification methods. Models were conducted separately for each outcome with the participants’ sex and household income included as covariates and with mental health change as the exposure. Data were analyzed July 5, 2020.

## Results

The total participating sample included 9369 adolescents, including 4665 (48.1%) girls and 6014 participants (81.9%) who were born in England. Among the total cohort, mean (SD) body fat percentage at age 14 years was 21.7% (9.2%) (Table 1). A total of 6106 participants had no reported mental health problems at either time point, 693 participants had parent-reported problems at age 7 years only, 2197 participants had self-reported problems at age 14 years only, and 373 participants had mental health problems at ages 7 and 14 years. Behavioral characteristics by mental health category are reported in Table 2. In adjusted regression models (Table 3), adolescents who self-reported mental health problems at age 14 years only were less likely to achieve 9 hours or more of sleep (odds ratio [OR], 0.39; 95% CI, 0.34-0.45), were less likely to consume fruit (OR, 0.55; 95% CI, 0.46-0.65) and vegetables (OR, 0.66; 95% CI, 0.52-0.83), and used more social media (*b* = 0.62; 95% CI, 0.49-0.75) compared with participants with no reported mental health problems at either time point (Table 3). Similar findings were observed for those with mental health problems at both time points for sleep (OR, 0.68; 95% CI, 0.51-0.90), fruit (OR, 0.39; 95% CI, 0.26-0.58) and vegetable consumption (OR, 0.57; 95% CI, 0.35-0.91), and social media use (*b* = 0.63; 95% CI, 0.34-0.91).

**Table 1.** Participant Characteristics From the Millennium Cohort Study at Ages 7 and 14 Years

Characteristic	Participants, No. (%)^a^				
	With no reported mental health problems (n = 6106)	With mental health problems			Total (N = 9369)
		Parent-reported at age 7 y only (n = 693)	Self-reported at age 14 y only (n = 2197)	At ages 7 and 14 y (n = 373)	
Sex					
Boys	3407 (57.6)	477 (70.9)	663 (30.8)	157 (45.8)	4704 (51.9)
Girls	2699 (42.4)	216 (29.1)	1534 (69.2)	216 (54.2)	4665 (48.1)
Country of birth					
England	3847 (81.1)	485 (84.5)	1424 (82.7)	258 (85.4)	6014 (81.9)
Wales	872 (5.1)	90 (4.8)	355 (5.6)	46 (3.3)	1363 (5.1)
Scotland	738 (9.3)	54 (6.3)	225 (8.0)	33 (7.9)	1050 (8.6)
Northern Ireland	649 (4.6)	64 (4.4)	193 (3.7)	36 (3.4)	942 (4.3)
Household income					
First quantile	699 (13.1)	190 (33.8)	253 (12.6)	106 (29.8)	1248 (15.6)
Second quantile	833 (15.5)	156 (21.7)	368 (19.7)	91 (28.7)	1448 (17.7)
Third quantile	1263 (20.1)	140 (20.1)	455 (21.5)	80 (20.5)	1938 (20.5)
Fourth quantile	1596 (24.1)	102 (12.3)	572 (23.3)	63 (13.9)	2333 (22.4)
Fifth quantile	1715 (27.1)	105 (12.2)	549 (22.8)	33 (7.1)	2402 (23.8)
% Body fat, mean (SD)	20.5 (8.9)	20.8 (10.2)	24.6 (8.7)	24.2 (9.7)	21.7 (9.2)

^a^ Counts are unweighted, and percentages are weighted. Means and SDs are weighted.

**Table 2.** Health Behaviors at Age 14 Years by Mental Health From Childhood and Adolescence

Health behavior	No. (%)^a^				
	No mental health problems	With mental health problems			Total population
		Parent-reported at age 7 y	Self-reported at age 14 y	At ages 7 and 14 y	
Sleep, h					
<9	2258 (37.3)	254 (36.9)	1289 (60.6)	190 (49.2)	3991 (43.2)
≥9	3848 (62.7)	439 (63.1)	908 (39.4)	183 (50.8)	5378 (56.8)
Fruit consumption					
No	423 (6.9)	63 (9.2)	254 (11.4)	59 (17.5)	799 (8.7)
Yes	5683 (93.1)	630 (90.8)	1943 (88.6)	314 (82.5)	8570 (91.3)
Vegetable consumption					
No	397 (6.6)	65 (10.1)	209 (9.1)	45 (13.0)	716 (7.8)
Yes	5709 (93.3)	628 (89.9)	1988 (90.9)	328 (87.0)	8653 (92.2)
Soft drink consumption					
No	1996 (30.6)	167 (22.1)	748 (31.7)	87 (22.5)	2998 (29.7)
Yes	4110 (69.4)	526 (77.9)	1449 (68.3)	286 (77.5)	6371 (70.3)
Social media use, hr/wk					
<0.5	1270 (20.6)	196 (25.6)	272 (11.2)	72 (17.0)	1810 (18.7)
0.5 to <1	978 (15.5)	101 (13.6)	248 (11.7)	34 (7.8)	1361 (14.0)
1 to <2	1117 (18.4)	122 (17.7)	324 (13.9)	50 (14.7)	1613 (16.9)
2 to <3	1022 (16.6)	91 (13.9)	322 (15.3)	41 (10.6)	1476 (15.7)
3 to <5	867 (14.0)	62 (10.5)	375 (17.4)	47 (14.0)	1351 (14.5)
≥5	852 (14.9)	121 (18.6)	656 (31.3)	129 (35.9)	1758 (20.1)

^a^ Counts are unweighted, and percentages are weighted.

**Table 3.** Regression Models for Mental Health From Childhood to Adolescence and Outcome Health Behaviors at Age 14 Years Adjusted for Sex, Country of Birth, and Household Income

Mental health problems	Odds ratio (95% CI)				Social media use, *b* (95% CI)^c^
	Sleep^a^	Weekly dietary consumption^b^			
		Fruit	Vegetable	Soft drink	
None	1 [Reference]	1 [Reference]	1 [Reference]	1 [Reference]	1 [Reference]
Parent-reported at age 7 y^d^	1.10 (0.89 to 1.35)	0.86 (0.60 to 1.22)	0.83 (0.57 to 1.20)	1.15 (0.88 to 1.48)	−0.09 (−0.29 to 0.11)
Self-reported at age 14 y^e^	0.39 (0.34 to 0.45)	0.55 (0.46 to 0.65)	0.66 (0.52 to 0.83)	1.05 (0.93 to 1.18)	0.62 (0.49 to 0.75)
Reported at ages 7 and 14 y	0.68 (0.51 to 0.90)	0.39 (0.26 to 0.58)	0.57 (0.35 to 0.91)	1.25 (0.91 to 1.71)	0.63 (0.34 to 0.91)

^a^ Likelihood of receiving 9 hours of sleep or more, as per sleep recommendations for this age group.

^b^ Categorized as a binary yes or no variable, with yes as the outcome.

^c^ Hours of use per week categorized as 1, indicating none; 2, less than half an hour; 3, half an hour to less than 1 hour; 4, 1 hour to less than 2 hours; 5, 2 hours to less than 3 hours; 6, 3 hours to less than 5 hours; 7, 5 hours to less than 7 hours; and 8, 7 hours or more.

^d^ Reported using the Strengths and Difficulties Questionnaire.

^e^ Reported using the Short Mood and Feelings Questionnaire.

## Discussion

### Principal Findings

This cohort study in a nationally representative sample of young people in the UK found that, compared with adolescents without reported mental health problems, self-reported mental health problems at both time points and in adolescence only were negatively associated with adolescents’ adherence to sleep guidelines and fruit and vegetable consumption and positively associated with increased social media use. To our knowledge, this is the first nationally representative longitudinal study of young people in the UK to adopt a multiple lifestyle behavioral perspective, which assessed the associations of parent- and self-reported mental health with multiple health behavioral outcomes. Our study was strengthened by the nationally representative sample and adjustment for known confounding demographic and health characteristics of young people.

Our findings align with those previously reported in the same data cohort identifying an association of nonadherence to recommendations for sleep with increased risk of depressive symptoms,^24^ and reduced odds of depressive symptoms observed among participants who regularly achieve physical activity guidelines of 60 minutes per day in childhood and adolescence.^25^ These findings are particularly important given the large burden of physical noncommunicable diseases attributable to lifestyle behaviors and the need for a strengthened understanding of the interconnected relationships between mental and physical health experiences of young people. However, we did not find an association of parent-reported mental health problems at age 7 years only with later health behaviors. This was inconsistent with our hypothesis of an association between mental health problems and adverse health behavioral outcomes.

### Findings in the Context of Previous Evidence

Not achieving the recommended 9 hours sleep was associated with self-reported mental health problems at both time points and at age 14 years only. The reciprocal association of sleep problems and depression has been established in large adolescent cohorts,^26,27^ additionally strengthened by depression treatment interventions through which sleep has been shown to improve as a residual.^28^ Proposed mechanisms include improved immune functioning resulting from quiet, deep sleep and enhanced learning, memory, and emotional health through rapid eye movement sleep.^29^ Critically, insomnia has also been established as a risk factor associated with development of future depression in adults and adolescents.^29^ A 2016 meta-analysis^30^ reported that individuals with insomnia experienced a more than 2-fold risk of developing depression compared with those who do not experience insomnia. There have been subsequent calls for prevention of depression to be a primary aim for individuals experiencing sleep disturbance, in addition to insomnia being considered a symptom of depressive disorders.^31^ While having parent-reported mental health problems at age 7 years only was not associated to sleep at age 14 years in our study, it is possible that the time of onset of depression and underlying symptoms are more likely to have occurred with the onset of adolescence. We also note that the characteristics of sleep behaviors, such as quality of sleep and timing, as opposed to purely duration, may have important implications for mental health and vice versa.^32,33^ Further examination of the association of mental health during adolescence with sleep behavior is warranted, as is the consideration of the utility of public health sleep guidelines that may overlook such complexity with a cutoff that may be considered arbitrary.

Fewer individuals who self-reported mental health problems at both time points and at age 14 years only were fruit and vegetable consumers, compared with individuals without mental health problems at either time point. This is consistent with the wider nutritional psychiatry evidence base that suggests an association of mental health problems with diet quality.^34,35,36,37^ It was notable that having parent-reported mental health problems in early life only was not associated with fruit, vegetable, or soft drink consumption, as these indicators are regularly used as proxies for overall diet quality in the epidemiological literature. There have been criticisms that using individual intakes of single food items is unlikely to reflect an overall dietary pattern and that it is consumption patterns that interact over time through various inflammatory mechanisms that contribute to mental and brain health.^34,38^ It is highly probable that our dietary assessment inadequately captured such mechanisms. This demonstrates the need for including comprehensive dietary intake measures in large population-representative surveys, with such methods holding far greater insight and implications for population health inferences.

The association of increased social media use among individuals with depression, as found in this study, has previously been established through meta-analysis, and has previously been reported in the MCS.^39^ Although the underlying factors of this association have yet to be determined, it has previously been shown that social interactions in the online world mirror many of the same psychological and neurological processes involved in real-world social interaction.^40^ Whereas this could present social media as a coping tool for mental health, it also introduces the potential for certain artificial aspects on online social networks to evoke adverse effects on mental health.^40^ For instance, the tendency for unrealistic upward comparisons in online social networks can have a negative influence on self-esteem.^41^ Additionally, individuals with pre-existing mental health conditions may be more prone to bullying and perceived victimization online or to seek out depressive or upsetting content, resulting in reinforcing spirals of poor mental health.^41^ However, the research into the connection between social media use and mental health is still in its infancy, and further longitudinal and experimental studies are required to establish the causality, strength, and mechanisms of the associations observed to date.^42^

### Limitations

A major limitation of our study is that we assessed parent-reported mental health problems at age 7 years and self-reported depressive symptoms at age 14 years, and these measures may have failed to capture some individuals experiencing mental health problems with discordance between reporting methods previously established.^43^ As depression can be episodic, it is possible that those with mental health problems present at age 14 years may have experienced depressive symptoms earlier in life. The participation numbers for the defined mental health categories are imbalanced, and we acknowledge that there is potential for type 1 and type 2 error due to this imbalance. We also recognize that our 2 mental health measures, while they have shown consistency in measuring mental health at the population level, were likely to capture different constructs, and the extent to which they can be compared is limited. For example, while the SDQ (parent-reported at age 7 years) aims to capture both internalizing and externalizing problems during a 6-month period, the SMFQ (self-reported at age 14 years) focuses primarily on internalizing symptoms during the preceding 2 weeks. Despite this, our findings relate predominately to the experiences at age 14 years, which extends the evidence base to date, as described. We also acknowledge the limitations in applying cutoff values to the mental health measures, and assessing through a continuous measure of symptoms may have revealed further insight into these associations. However, we identified the public health and clinical relevance of cutoff values in identifying participants experiencing problems that were likely to interfere with daily life and accepted that this method was appropriate for the aims of this study. While the participant numbers across mental health groups appear imbalanced, we note the magnitude and direction of associations substantiate the unique health behavior status of those with self-reported mental health problems at age 14 years only.

Lifestyle behavioral factors were all self-reported which were potentially influenced by social desirability, recall, and other biases. Further limitations were that the sleep item asked for time in bed as opposed to sleep duration, and this may have affected the accuracy of this indicator. We acknowledge the likelihood of clustering of health behaviors that is likely to have occurred. We were also unable to control for health behaviors at age 7 years owing to data unavailability; thus, our findings may have been limited without these data. Our analysis did not account for other important potential confounders, such as adverse childhood experiences and family history of mental health problems, which likely contribute to both mental health problems and health behavioral factors. While outside the scope of this research, we suggest this is an important area for future research.

## Conclusions

This cohort study found that the presence of self-reported mental health problems at ages 7 and 14 years and at age 14 years only was associated with some health behaviors at age 14 years, including sleep, fruit and vegetable consumption, and social media use. These findings are particularly important for public health and clinical practice, given that health behaviors can deteriorate and become habitual during adolescence, and it is also a known time for the first emergence of mental health problems that continue into adulthood.
